# Design and preclinical feasibility of a pediatric heart valve stent that spontaneously adapts to growth via a spring mechanism

**DOI:** 10.1126/sciadv.adw4669

**Published:** 2026-02-18

**Authors:** Giselle Ventura, Masaki Kajimoto, Mossab Saeed, J. Scott Malloy, Ian McGinty, Lyubomyr Bohuta, Kevin A. Charette, Kyle Bilodeau, Gianna Dafflisio, Shannen B. Kizilski, Peter E. Hammer, John P. Carney, David W. Sutherland, Baturalp Arslan, Daniel F. King, David J. Carter, Sitaram M. Emani, Michael A. Portman, Corin Williams

**Affiliations:** ^1^ The Charles Stark Draper Laboratory Inc., Cambridge, MA, USA.; ^2^ Seattle Children’s Research Institute, Seattle, WA, USA.; ^3^ Boston Children’s Hospital, Boston, MA, USA.; ^4^ Experimental Surgical Services Laboratory, University of Minnesota, Minneapolis, MN, USA.

## Abstract

Congenital heart valve defects often require surgical intervention for survival. Now, there are no valve prosthetics on the market that are designed for babies and toddlers who grow rapidly. Recent advancements in expandable valves hold promise for these young patients but currently require invasive balloon catheter procedures for device expansion. Here, we describe the design and preclinical feasibility of a pediatric heart valve stent that spontaneously adapts to growth via a springlike mechanism imparted by the superelastic properties of nitinol. Studies in piglets demonstrated appropriate constraint of the device to small diameters (8 to 9 millimeters) upon implantation, acute valve function, and the ability of the stent to proportionately expand with growth up to 13 millimeters after 6 weeks without subsequent interventions after implantation. We expect that the growth-adaptive stent concept could be broadly leveraged to eliminate surgeries and invasive procedures as young patients grow.

## Teaser

A pediatric heart valve stent that spontaneously “grows” could reduce surgeries and invasive procedures for children.

## INTRODUCTION

Congenital heart defects (CHDs) account for nearly one-third of all major congenital defects and are a leading cause of morbidity and mortality in children (*1*). Nearly 1% of babies born each year will have a CHD, equating to about 40,000 annually in the US and over 1 million worldwide (*2*). Although surgical advancements over the past few decades have led to increased survival, nearly half of CHD-related deaths occur within the first year of life (*3*). Valve defects occur in 20 to 30% of patients with CHD and often require surgical intervention for survival (*4*), typically within the first few days to months after birth. Any of the four cardiac valves may be defective in a CHD, with the pulmonary valve (PV) being one of the most affected (*2*) due to conditions such as isolated pulmonary stenosis (*5*) and tetralogy of Fallot (*6*). It has been reported that as many as 40% of patients with CHD will require PV replacement at least once in their lifetime (*7*). Unfortunately, valve replacement options remain limited for young pediatric patients because the market has been driven primarily by adult needs, where the mean age at valve replacement is around 65 years old (*8*). These commercially available devices are designed to operate at discrete sizes and are large, with diameters typically in the range of 18 to 36 mm (*9*). As of 2025, the smallest valve on the market is the Abbott Master’s HP mechanical valve for aortic or mitral valve replacement, with a diameter of 15 mm (*10*). Although this device has been a lifesaving option for neonates and infants, it must be surgically replaced once outgrown within a few years (*11*). For PV replacement, the bioprosthetic Medtronic Melody Valve currently has the smallest diameter at 16 mm (*4*, *12*). However, commercially available bioprosthetic valves are still too large for infants and toddlers and are used off-label in this patient population, often with heavy modifications to the device (*13*–*15*). Consequently, a child who undergoes their first valve replacement surgery under the age of 2 is currently destined to have as many as five open-heart surgeries before adulthood as these devices fail or are outgrown (*16*–*18*). There remains a clear unmet clinical need for valve replacement options in patients under 20 kg (*12*) who are typically younger than 4 to 6 years old (*19*).

Although the ideal valve replacement strategy in young children with CHD remains elusive, the following characteristics are broadly desired and are also particularly unique to pediatric valve design: (i) small device dimensions to fit pediatric anatomy, (ii) functionality of the valve at small diameters, and (iii) the ability of the device to adapt to the child’s growth while maintaining valve function. Cardiac valves are as small as 7 to 10 mm at birth and roughly double in diameter by age 5 to 6 years old, with at least a tripling in diameter over the human life span (*13*). A number of pioneering and creative solutions have been attempted in recent years to address the challenge of growth. Tissue engineering is especially promising for pediatric patients as a solution that could provide somatic growth through the use or creation of living tissue (*20*). Although many tissue-engineered heart valves have been developed in the past 20 years (*21*), one of the most notable recent advancements was by Syedain *et al.* demonstrating a 19- to 25-mm diameter increase over 52 weeks in a growing lamb model (*22*). Despite exciting progress in the field, tissue-engineered heart valves have not yet achieved commercialization and the regulatory pathway for tissue-engineered products remains unclear (*7*). An alternative approach to achieving growth is the concept of an expandable device, which was first demonstrated clinically with the Medtronic Melody Valve (*15*, *23*). One of the serendipitous features of the Melody Valve is its utilization of a bovine jugular venous valve, which can operate across multiple diameters (*15*, *24*, *25*). The venous valve enables the device to maintain function at a compressed diameter as small as 9 mm and be expanded via a balloon catheter during the child’s growth (*25*). However, limitations include the requirement to make heavy modifications to the device to fit small patients (*14*) and reported propensity for infection compared to other bioprosthetic valves (*26*). Nevertheless, the Melody Valve paved the way for several groups to further develop the expandable valve concept as a promising solution for pediatric patients (*27*–*31*). One key example is the polymeric Autus Valve, which mimics venous valve geometry, can be balloon expanded from 12- to 24-mm diameter (*27*), and initiated first-in-human trials in 2021 (*32*). However, expandable valves still face limitations in terms of size and, in particular, the need for transcatheter balloon expansion at discrete time points during growth (*15*), which is an invasive procedure involving anesthesia, hospitalization, and risk of damage or infection of the valve with each intervention (*33*, *34*). Ideally, an expandable pediatric heart valve could be achieved without invasive methods via spontaneous and proportional adaptation to growth.

**Fig. 1.**
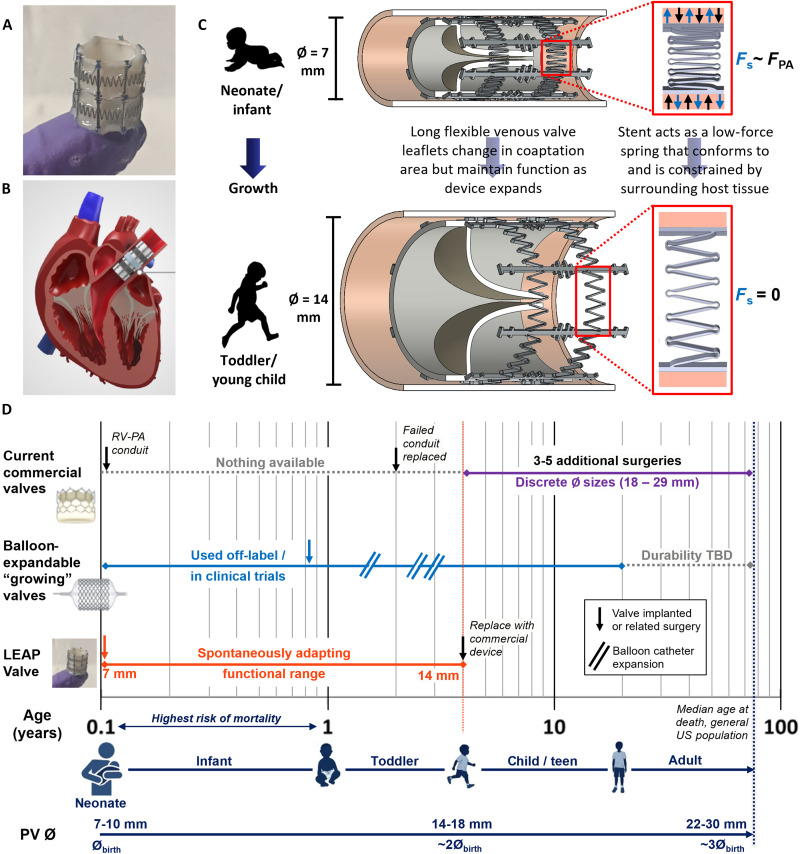
Growth-adaptive LEAP Valve concept. (**A**) LEAP Valve prototype device used in animal studies, fully expanded configuration (14 mm in diameter, 15 mm in length), on a fingertip. (**B**) The device is designed to be implanted during open-heart surgery for PV replacement, positioned within the main PA. (**C**) Cross-sectional schematic demonstrates how the stent adapts its diameter (Ø) while the expandable valve maintains function with growth. The LEAP Valve is implanted in a compressed configuration in neonates or infants (top) and then expands up to twofold as the child grows (bottom). Renders and insets of stent elements illustrate the function of the spring mechanism. (**D**) Comparison of LEAP Valve to current PV replacement technologies for children with valve defects, emphasizing the age range of critical unmet need (birth to 4 years old; orange vertical dotted line). Commercial valves are designed for older patients, with discrete diameter sizes available at 18 mm and larger (*9*). Young patients typically receive a RV-PA conduit soon after birth, which fail within about 2 years (*47*, *48*), leading to repeat surgeries before adulthood (*49*). Emerging technologies include balloon-expandable devices, which are currently used off-label in young children with heavy modifications (*15*) or are in clinical trials (*32*). Balloon-expandable valve implantation and transcatheter interventions are median time points reported in young children (*15*). The LEAP Valve would fill a technology gap by providing continuous function over a diameter range of 7 to 14 mm without the need for additional interventions after implantation, eliminating one or more invasive procedures currently required. Valves diameter estimates are from multiple references (*13*, *71*, *72*). Pediatric subpopulations follow Food and Drug Administration (FDA) definitions (*73*). TBD, to be determined. Portions of (D) created in BioRender. Williams, C. (2025) https://BioRender.com/kr12iw7.

Here, we present the design, predictive modeling, acute bench tests, and early preclinical feasibility studies of the Low-force Expanding/Adaptable Pediatric (LEAP) Valve, which spontaneously and noninvasively adapts to growth up to a doubling in outer diameter from 7 to 14 mm (Fig. 1A). The initial indication for the LEAP Valve is PV replacement (Fig. 1B), designed to fit the anatomy of neonates and infants. Given that the venous valve has already been proven in other contexts as described above, we focused this work on developing and characterizing the growth-adaptive stent. The key innovation of the stent is that it is designed to function as a low-force spring by exploiting the superelastic properties of nitinol (*35*), thus providing the expansion mechanism for the venous valve contained within. The device is implanted in a compressed configuration in which the stent conforms to and is constrained by the surrounding tissue at the implantation site, leading to a balance of the outward radial force exerted by the stent and restraining force of the host tissue (Fig. 1C, top). The stent then proportionally expands via continued exertion of its low outward radial force as the native tissue grows, until the stent reaches the zero-stress state at its fully expanded diameter of ~14 mm (Fig. 1C, bottom). The long-term vision of this work is to address an unmet clinical need and technology gap for the population of patients with CHD by providing a PV replacement for children with valve diameters <15 mm (neonates, infants, and toddlers) who grow rapidly in the first 4 to 6 years of life (Fig. 1D). The utilization of the growth-adaptive stent in pediatric valve prosthetics could reduce the number of surgeries and/or invasive procedures that these young patients typically endure for heart valve replacement using current state-of-the-art commercial devices or emerging balloon-expandable devices (Fig. 1D).

## RESULTS

### Concept, design, and in vitro performance of the growth-adaptive LEAP Valve stent

#### Device concept

The LEAP Valve was designed to address a clinical need and technology gap for heart valve replacement in pediatric patients under 5 years old and was named for its intended operation (Fig. 1). The core technologies of the LEAP Valve are (i) the growth-adaptive stent designed to spontaneously expand with growth via a springlike mechanism (*35*) and (ii) a biological venous valve that can maintain function over a doubling in diameter (*28*). The current design of the LEAP Valve stent has a functional outer diameter range of 7 to 14 mm or twofold expansion capability, in line with a doubling in valve diameter from birth to age 5 to 6 years old (*13*). The device is designed for PV replacement during open-heart surgery, with the ideal implantation site in the main pulmonary artery (PA) (Fig. 1B). Dimensional and functional requirements were guided by preclinical data and clinical experience (*28*, *36*, *37*). Specifically, the diameter range was selected to fill the current commercial technology gap and unmet clinical need for infants and toddlers with valve diameters less than 15 mm. The device length was restricted to 15 mm to fit the anatomy of young children and the preclinical animal model selected for implantation studies (*27*, *36*), as well as to sufficiently accommodate the commissural height of the venous valve. We selected the venous valve as the expandable valve for our device due its ability to function over multiple diameters, which has been demonstrated computationally (*37*), preclinically (*27*, *28*), and clinically (*14*, *15*) for balloon-expandable heart valve designs.

The concept for LEAP Valve implantation is to deliver the device in a compressed configuration during open-heart surgery in early life as young children with valve defects often require surgical repair of other structural heart defects, such as truncus arteriosus, transposition of the great arteries with pulmonary stenosis, and tetralogy of Fallot with pulmonary atresia. Often these patients require biventricular repair during the neonatal period using right ventricle (RV)–PA conduits with a diameter of 8 to 10 mm; the LEAP Valve would be a growth-adaptive alternative to this approach as a PV replacement within the native PA. At the smallest initial diameter of 7 mm, there will be substantial overlap of the valve leaflets (Fig. 1C, top). The compressed stent will act like a spring that exerts low chronic outward force, which is constrained by the strength of the surrounding native tissue. Balancing the force of the stent with the native tissue at the implantation site will prevent the device from fully expanding upon implantation. As the child grows, the device spontaneously expands due to the springlike behavior of the stent. The valve contained within the stent expands with it: The leaflets lose overlap area but maintain coaptation and thus maintain function (Fig. 1C, bottom). Once the LEAP Valve reaches its maximum diameter of 14 mm, the stent will be in its zero-stress state and no longer spontaneously expand. At this point, the patient could receive a larger surgical or transcatheter device.

#### Stent design and performance

**Fig. 2.**
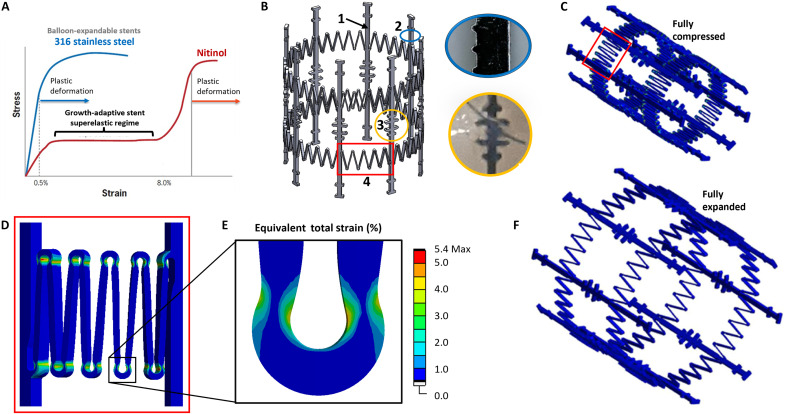
Growth-adaptive LEAP stent design and simulation. (**A**) Example stress-strain curves of two common stent materials: stainless steel and nitinol. Stainless steel balloon-expandable stents permanently and plastically deform when expanded. The nitinol LEAP stent passively expands over a large range of diameters by exploiting nitinol’s superelastic regime. (**B**) Render of the device showing key features of the stent. (1) Longitudinal struts provide stability and maintain a constant device length of 15 mm with changes in diameter (black arrow). (2) Small clocking features allow for detailed dimensional characterization for quality control (blue circle and inset; strut width: 400 μm). (3) Suture features along the struts provide anchoring points for valve attachment to the stent during device assembly (yellow circle and inset). (4) Springs provide the growth-adaptive function of the stent (red box), further detailed in (C) and (D). (**C**) Finite element simulation showing the concentration of strains in the spring bends when the stent diameter is compressed to 7 mm in diameter (red box). (**D** and **E**) Example strain plots for a single spring element and single spring bend show the maximum equivalent strain of 5.4%, below nitinol’s superelastic limit of 8%. (**F**) Simulation of the fully expanded stent in its zero force state.

To achieve the desired spring behavior of the growth-adaptive stent, we chose to exploit the superelastic properties of nitinol, a common material for cardiovascular stents (*38*). This property permits elastic deformation and recovery up to ~8 to 10% strain, unlike balloon-expandable stents, which must be plastically deformed (Fig. 2A). The final stent design used in the animal studies reported in this work had several key features, as shown in Fig. 2B. First, to achieve a constant device length of 15 mm, eight equally spaced longitudinal struts were implemented in the design. This approach avoids drastic changes in device length with diameter that are common in other stent designs (e.g., closed cell), which consequently could affect venous valve function. Microscopic clocking features were included on the struts for detailed dimensional analysis and quality control of the manufactured stents (*39*). In addition, suturing features for valve attachment to the stent were included at discrete locations along each strut and were designed to minimize suture slippage.

The most critical feature of the stent design was the series of springs elements, which were interspersed between the longitudinal struts. The springs had to meet the following criteria: (i) to not exceed a collective maximum radial outward force of 16 N, to avoid overexpansion of or damage to the surrounding tissue upon implantation (*40*, *41*), and (ii) to enable a twofold change in stent diameter while staying within nitinol’s superelastic strain regime <8%. Stent designs were developed in SolidWorks, and the strain experienced during stent compression and expansion was modeled in ANSYS. Spring elements were designed with a depth-to-width ratio of >1 to promote preferential bending and minimize buckling (*35*). Finite element analysis (FEA) revealed that the maximum strains at the fully compressed diameter of 7 mm were experienced in the spring bends (Fig. 2, C and D). The maximum strain at these features was 5.4% (Fig. 2E), sufficiently within the superelastic strain limit of 8%.

**Fig. 3.**
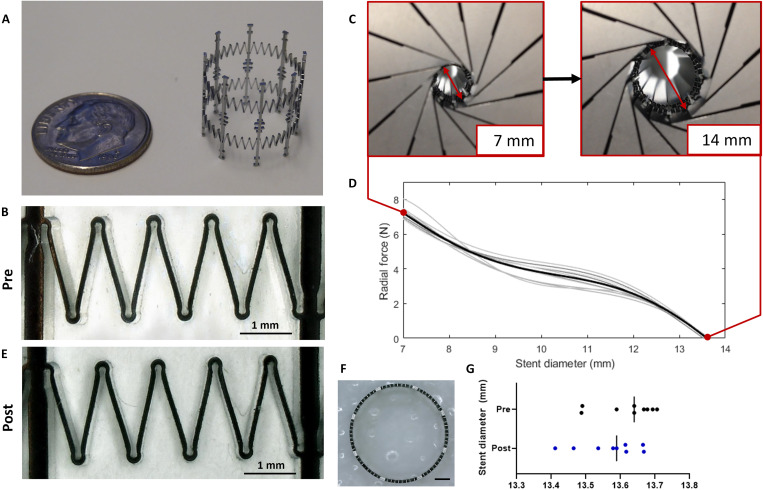
Manufacturing and radial force testing of growth-adaptive stents. (**A**) Growth-adaptive stent in the expanded state next to a dime for size comparison. (**B**) Close-up of spring features on manufactured stent, pretesting. (**C**) Stent radial force as a function of stent diameter was measured by a RX550/650 radial compressor as a stent was unloaded from the 7-mm-diameter compressed state to the ~14-mm-diameter expanded state. (**D**) Force-diameter unloading curves of the stents selected for use in animal studies (*N* = 10). Individual stents are shown in gray, and the mean unloading behavior is shown in black. (**E**) Same spring elements shown in (B), post–radial force testing. (**F**) Representative top view image of the stent used for diameter measurements. Scale bar, 2 mm. (**G**) Measurements of stent diameter pre– and post–radial force testing.

Manufactured stent prototypes underwent radial force testing to measure performance. Dimensional characterization of our growth-adaptive stent design in a previous work demonstrated that the small and complex features of the stent springs could be reliably manufactured (*39*) (Fig. 3, A and B). Radial force testing demonstrated consistent performance of the stent design selected for animal studies, with an average maximum force of 7.21 ± 0.25 N at the fully compressed diameter of 7 mm (Fig. 3, C and D, and table S1). This maximum force was well below the threshold identified in the prior literature cited above. In addition, we determined whether plastic deformation occurred in the manufactured prototypes, given the large deformations (100% change in stent diameter) during radial force testing. There was a negligible, nonsignificant reduction in stent diameter (<0.5%, *P* value = 0.23) after radial force testing, indicating that the stents maintained their superelastic properties even with large changes in diameter, as intentionally designed (Fig. 3, E to G).

#### Implantation model

Although 16 N has been identified as a high chronic outward force threshold for vascular tissue damage (*40*, *41*), this value is highly dependent on the mechanical properties of the specific tissue at the implantation site. To this end, we modeled the interaction between the growth-adaptive stent and the specific implantation site for our animal studies to estimate the predicted change in PA diameter that would be caused by the outward radial force of the stent. We used empirical data from our previous work, which characterized the mechanical properties of the main PA of Yucatan piglets (*36*), and radial force data from our manufactured stent prototypes. By deriving the applied force on the PA wall, we estimated the equilibrated force and resulting deformation range of the PA diameter, assuming an initial stent outer diameter and PA lumen diameter of 7 mm. This diameter represents the worst case scenario, in which the stent would exert its maximum outward radial force of ~7 N when initially placed within the PA (fig. S1). Starting from an initial diameter of 7 mm, the model predicted expansion to 7.9 mm (range: 7.7 to 8.7 mm), corresponding to an average diameter change of 13% (range: 10 to 24%). This occurred once the outward radial force of the stent balanced with the restraining force of the PA tissue at ~5.5 N (4.8 to 6 N). Although there is no direct correlation for our device, studies of nitinol stent oversizing for other indications suggest that a <40% diameter increase due to outward radial force is preferred to minimize tissue damage or adverse remodeling (*42*, *43*). Therefore, the amount of predicted strain on the native tissue from our growth-adaptive stent was deemed acceptable. Collectively, our modeling and empirical results indicated a successful stent design that could be tested in animals.

### Development of the assembled LEAP Valve device

#### Valve selection and device

**Fig. 4.**
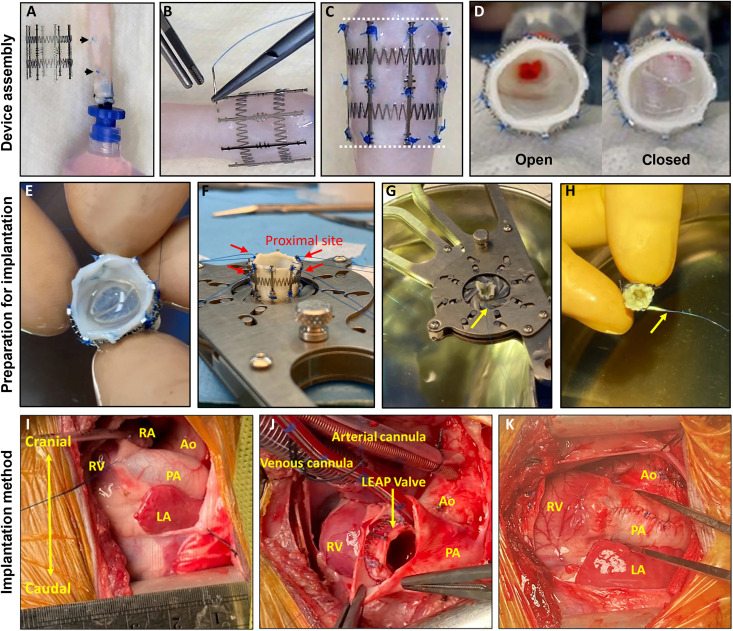
Device assembly and implantation strategy. (**A**) The valve is identified within the vein graft and demarcated by two sutures (black arrowheads). (**B**) The fully expanded stent is placed over the graft, and the valve is inflated with saline until flush against the inner surfaces of the stent. The valve is attached to the stent via suturing at the suture features along the stent struts. (**C**) Example of an integrated LEAP Valve device after all sutures have been placed. The excess graft tissue outside of the stented region is then trimmed (dotted lines). (**D**) Valve function is checked by applying forward and back pressure on the valve to open and close the leaflets, respectively. (**E**) Immediately before implantation, valve competency is checked again. (**F**) Anchoring sutures are attached to the proximal end of the stent (red arrows). (**G**) The device is then crimped down to 7 mm in diameter with the radial compressor tool, and a restraining suture is placed around the middle of the stent. (**H**) Crimped device at 7 mm in diameter, ready for implantation. The restraining suture “tail” is indicated by yellow arrows in (G) and (H). (**I**) Surgical procedure for implantation: The chest is opened, and the PA is identified. (**J**) The piglet is placed on CPB, and an incision is made in the main PA. Before implantation of the LEAP Valve, the native PV leaflets are resected. The crimped LEAP Valve is then implanted in the main PA with the proximal end of the device anchored at the native PV annulus. (**K**) Immediately before full closure of the PA, the restraining suture is released to allow the device to conform to the surrounding tissue.

Balloon-expandable venous valve designs have been previously tested for larger size ranges (*15*, *23*, *27*, *28*) but not in the 7- to 14-mm-diameter range of interest for our device. We first considered two different venous valve sources that were commercially available and had demonstrated clinical feasibility: (i) the Contegra Valve, a bovine jugular venous valve used for reconstruction of the right ventricular outflow tract and the same valve source used in the Melody Valve, and (ii) the cryopreserved human femoral vein (HFV) graft (Artivion, formerly CryoLife), which has been used as an RV-PA conduit in young patients (*44*). We found that the Contegra Valve was generally too large to fit our stent and the three leaflets had restricted motion at compressed diameters. In contrast, the HFV was in the appropriate diameter range with a thin profile and bileaflet geometry that functioned at smaller diameters (table S2). Therefore, we chose the HFV as the graft source for the LEAP Valve. The HFV was attached to the growth-adaptive stent using sutures as shown in Fig. 4 (A to D). We designed a handheld radial compressor tool (fig. S2) to check acute LEAP Valve competency at multiple diameters after assembly (fig. S3). Devices that passed functional quality checks were used for in vitro hydrodynamic tests and animal studies.

#### In vitro hydrodynamic tests

Two devices were evaluated under neonatal and toddler flow conditions (table S3) while being mounted in slightly undersized holders. When deployed into the 8.5-mm neonatal-sized holder, the inner diameter of the unpressurized valve measured 6.0 ± 0.4 mm, whereas in the 14-mm toddler-sized holder, the inner diameter measured 9.4 ± 0.7 mm (fig. S4). Because of the high stiffness of the silicone holders, diameter measurements were below the predicted values for deployment within a PA of equivalent preoperative diameter, whereas pressure gradients were higher than expected. We focused on assessing valve regurgitation under these test parameters, which was mild across all test conditions for both devices: 4 to 7% with the exception of 14.6 ± 1.0% under neonatal normal cardiac output for one device. The complete in vitro results are summarized in table S4.

### Acute in vivo implantation showed competent valve function and appropriate constraint of the LEAP Valve at small diameters

#### Development of implantation strategy

We developed the surgical implantation strategy for the LEAP Valve in four piglets with the goals of achieving uniform device shape/function and minimizing time on cardiopulmonary bypass (CPB). As part of these studies, we explored device location and the number of anchoring sutures required to secure the device. The piglets ranged in weight from 5.0 to 10.6 kg, ages 36 to 69 days old (Table 1). We found that the main PA with a length of >15 mm was the ideal implantation site for the LEAP Valve and that two anchoring sutures at the proximal end of the device were sufficient to secure the LEAP Valve without device migration or paravalvular leak. Device implantation was successful in three of the four acute studies; one unsuccessful implantation was due to buckling of the device in piglet #2, caused by a short main PA (15 mm). The first three piglets were euthanized during surgery after implantation of the LEAP Valve and after acute functional measurements had been made. Piglet #4 was allowed to recover postoperatively for ~3 hours before euthanasia. The acute studies are described in further detail in the Supplementary Materials.

**Table 1.** Acute cohort measurements.

#	Age (days)	Weight (kg)	Main PA length (mm)	PA lumen diameter (mm)		Pressure gradient (mmHg)
				Preimplant	Postimplant	
1	36	5.5	16	9.0	9.7	~0
2	37	5.0	15	8.5	(Buckled)	(Buckled)
3	49	7.3	18	8.0	9.0	<2
4	69	10.6	20	11	12	<2

#### Acute LEAP Valve function and measurements

**Fig. 5.**
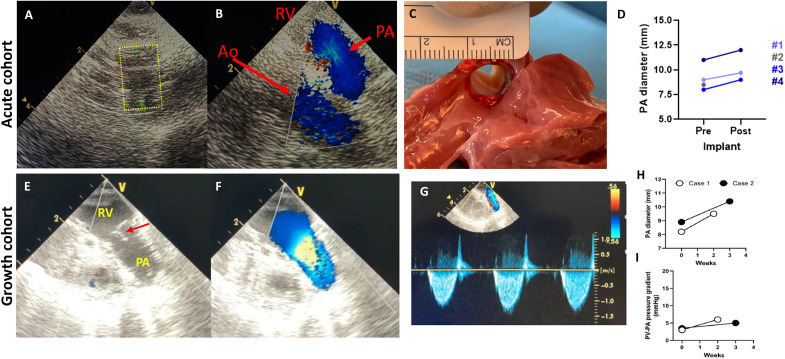
Acute LEAP Valve function and short-term growth studies. (**A**) Echocardiogram showing the LEAP Valve (yellow dotted outline) immediately after implantation. (**B**) Doppler flow image showing flow through the LEAP Valve immediately after implantation. (**C**) Explanted piglet heart with LEAP Valve after acute implantation, showing conformational device fit to the PA, constraint of the device to ~9 mm in diameter, and no acute thrombosis. (A to C) Data for piglet #3 in the acute cohort. (**D**) Graph of the PA diameter pre- and postimplant in the acute cohort measured by epicardial echo, indicating an average 9.8% increase in PA diameter upon implantation of the LEAP Valve, and the overall constraint of the device. Note that the device buckled upon implantation in piglet #2. (**E**) Echocardiogram image showing LEAP Valve leaflets (red arrow) at 2 weeks postimplantation. (**F**) Doppler flow image showing flow through the valve at 2 weeks. (**G**) The maximum pressure gradient was calculated using continuous wave Doppler through the implanted valve, according to the Bernoulli principle. Velocity, 1.22 m/s; pressure gradient, 6.0 mmHg in piglet #2 (2-week follow-up). (E to G) Piglet with 2-week follow-up. (**H**) Graph of the PA diameter measured by echo at implantation and terminal surgery for 2- and 3-week follow-up, indicating increased diameter with growth. (**I**) Transvalvular pressure gradient remained low immediately postimplantation (time 0) and at 2- and 3-week follow-up, indicating good valve function.

For the three successful acute, nonsurvival device implantations (piglets #1, #3, and #4), epicardial echocardiography during the surgical procedure showed good valve function, indicated by leaflet motion and flow through the valve without stenosis or paravalvular leaks (Fig. 5, A and B). Pressure measurements by catheter insertion demonstrated that the systolic pressure gradient between the RV and PA across the implanted valve was minimal (<2 mmHg) in these three piglets. Measurements of the PA diameter immediately pre- and postimplantation of the LEAP Valve showed, on average, a 9.8% increase in PA effective lumen diameter, aligned with our expectations from modeling stent interactions with the tissue (Fig. 5, C and D; Table 1; and fig. S1), indicating that the radial force of the growth-adaptive stent reached equilibrium with and was constrained by the stiffness of surrounding host PA tissue without fully expanding or rupturing the tissue. Macroscopic inspection of the explanted heart upon termination showed conformational fit of the LEAP Valve to the PA wall, with no acute thrombosis or clotting on the leaflets. Overall, these results indicated that the growth-adaptive stent design was within an appropriate force range to be constrained by the host tissue upon implantation. The next step was to assess the ability of the stent to actively adapt to growth without intervention in survival studies.

### Survival studies demonstrated the spontaneous growth-adaptive mechanism of the stent

#### Piglet growth projections

To estimate the implantation time frame for the LEAP Valve in growing piglets, we first examined the relationship between animal growth and PA diameter in 12 healthy Yucatan mini pigs (see the Supplementary Materials). The data provided by the pig vendor (Premier BioSource) showed ~5 kg of weight gain/month in 1- to 4-month-old Yucatan piglets. The pig housing data in our facility at the Seattle Children’s Research Institute after transportation from the pig vendor showed an average of 1.26 kg of weight gain/week at 1 to 2 months old. Growth in terms of weight gain per week or month was relatively linear in our age range of interest. We calculated the relationship between the PA diameter and body weight or age from the data of these pigs. Simple linear regression showed a positive correlation between PA diameter and body weight (fig. S5). On the basis of these data and the functional diameter range of the growth-adaptive stent (7 to 14 mm), the appropriate pig weight range for the LEAP Valve was estimated to be 4.5 to 15 kg. In other words, a tripling in body weight was expected to roughly correspond to a doubling in valve diameter. However, given our experience with the acute studies described above, we decided to use slightly larger piglets of ~7 kg (average PA lumen diameter of 8.7 mm, main PA length of >15 mm) to ensure that the main PA was of sufficient straight length to accommodate the LEAP Valve.

#### Short-term function after LEAP Valve implantation

We planned the LEAP Valve implantation for three piglets and euthanized one each on day of surgery (7.1 kg, 46 days old), at 2 weeks (initial weight: 6.1 kg; initial age: 53 days old) and 3 weeks (initial weight: 8.2 kg; initial age: 53 days old) postprocedure. The first piglet was euthanized shortly after LEAP Valve implantation surgery due to refractory ventricular arrhythmia, which was a procedural complication and not due to the LEAP Valve device. The two other piglets did not have any procedural complications or major adverse events during follow-up. We conducted terminal surgery at the predetermined postoperative follow-up time point (*N* = 1 each at 2 and 3 weeks after implantation, with respective weight gains of 2.8 and 1.4 kg). We note that piglet #3 with 3-week follow-up initially lost some weight due to minor illness after surgery but recovered after 3 days.

**Fig. 6.**
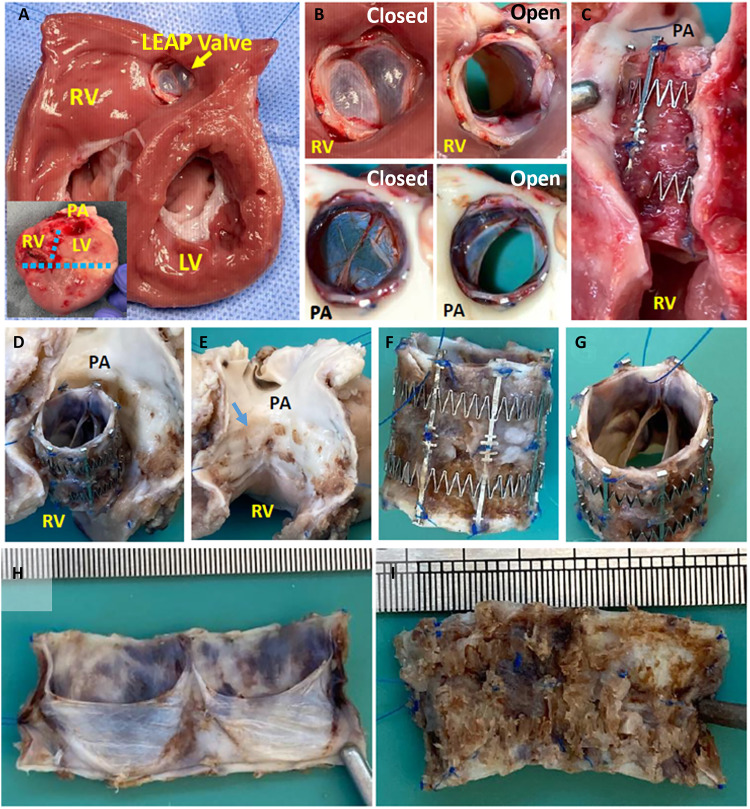
Explant of LEAP Valve at 2 weeks. (**A**) Cross section of the explanted heart viewed from the ventricular side. Inset shows section cuts. (**B**) View of the LEAP Valve from the RV side (top images) and PA side (bottom images), in open and closed states. (**C**) Partial removal of the PA around the LEAP Valve indicates good fitting of the device to the implantation site with no signs of tissue damage. (**D**) Partially extracted LEAP Valve. (**E**) Photo of the implantation site after removal of the LEAP Valve. Slight indentation from stent springs was observed (blue arrow). (**F**) Side and (**G**) top views of the explanted LEAP Valve. (**H**) View of valve leaflets and (**I**) adventitial side after removal of the stent. There were no obvious blood clots and no tearing/degradation of the leaflets. (A to C) Fresh. (D to I) After formalin fixation and hemosiderin staining.

Epicardial echocardiography (Fig. 5, E to I) at terminal surgery showed laminar blood flow through the implanted valve and smoothly moving, pliable leaflets without regurgitation. The inner diameter of the implanted valve increased by 1.3 to 1.5 mm after 2 to 3 weeks (Fig. 5H). Systolic pressure gradient between the RV and PA by a pressure catheter was <4 mmHg, and peak pressure gradient by Doppler examination of echocardiography was 5 to 6 mmHg (Fig. 5I). Macroscopic images of the resected tissue including the RV and PA showed the intact implanted valve without thrombus, in agreement with the echocardiographic examinations (Fig. 6). The inner PA wall that was attached to the implanted LEAP Valve was normal with slight pressure marks from the stent but no evidence of damage (Fig. 6E). Overall, these findings indicated that the LEAP Valve demonstrated good function and growth at early time points.

#### Longer-term outcomes

Given the encouraging results for early time points, we scheduled *N* = 3 animals that were euthanized at 4, 5, and 6 weeks after implantation of the LEAP Valve (piglets #4 to #6). The initial ages and weights of the piglets were as follows: 7.0 kg (50 days old), 6.3 kg (52 days old), and 6.8 kg (56 days old). During the postoperative follow-up, the piglets did not have any complications except minor surgical skin incision site infections. All piglets gained weight during the growth period: 6.6-kg gain after 4 weeks for piglet #4, 9.8-kg gain after 5 weeks for piglet #5, and 11.4-kg gain for piglet #6. Detailed growth and functional data are shown in Table 2 for the LEAP Valve implantation and terminal surgeries in the growth cohort (*N* = 6 animals total).

**Table 2.** Growth cohort measurements. Note that LEAP Valve lumen diameter (Ø) measurements were obtained by echo and are of the functional valve lumen not the stent. Stent OD was measured in situ within the explanted heart and PA (macroscopic photos at 2 and 3 weeks and x-ray images at 4, 5, and 6 weeks). AP, aortic pressure; PAP, pulmonary artery pressure; PG, pressure gradient between the RV and PA; LVP, left ventricle pressure; OD, outer diameter; RVP, right ventricle pressure; Wt, weight. Reported pressures are the systolic/diastolic (max/min) averaged over 10 cardiac cycles.

	LEAP Valve implantation surgery							Terminal surgery							Explant
Pig #	Age (days)	Wt (kg)	AP (mmHG)	PAP (mmHG)	RVP (mmHG)	LEAP Valve lumen ∅ (mm)	PG (mmHG)	Follow-up (weeks)	Wt (kg)	LVP or AP (mmHG)	PAP (mmHG)	RVP (mmHG)	LEAP Valve lumen ∅ (mm)	PG (mmHG)	Stent OD (mm)
1	46	7.1	79/52	15/12	12/8	9.3	0.9	–	–	–	–	–	–	–	–
2	53	6.1	83/53	16/13	14/7	8.2	3.0	2	8.9	71/41	14/10	12/5	9.5	6	10
3	53	8.2	69/43	14/10	13/7	8.9	3.5	3	9.6	67/6	22/15	26/4	10.4	5	10.6
4	50	7.0	96/43	18/15	20/8	9.4	3.0	4	13.6	81/6	20/14	52/4	8.8	28–30	12.8
5	52	6.3	79/55	13/10	12/6	8.6	1.0	5	16.1	84/4	19/15	46/5	9.7	23–25	11.9
6	56	6.8	79/40	16/12	20/6	8.5	2.3	6	18.2	76/4	20/16	35/5	9.3	13–15	13

**Fig. 7.**
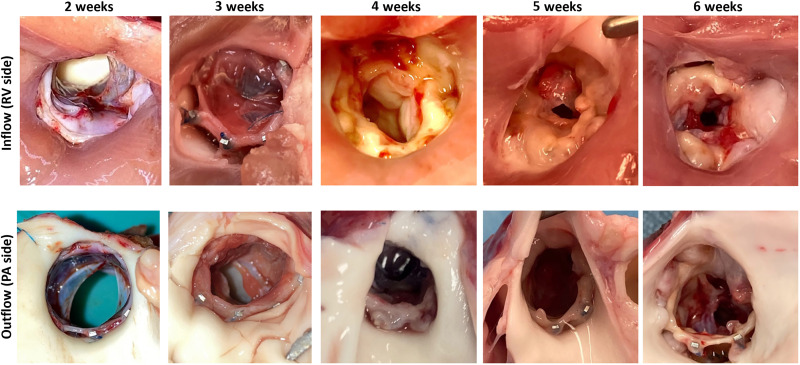
Macroscopic views of LEAP Valve inflow and outflow at endpoint. (**Top**) Inflow of the LEAP Valve in the explanted heart for 2- to 6-week follow-up. (**Bottom**) Corresponding images of the outflow of the device for each time point.

Epicardial echocardiography at 4- to 6-week follow-up showed turbulent flow inside of the implanted valve and higher transvalvular peak pressure gradients ranging 13 to 30 mmHg, with moderate pulmonary stenosis compared to the results at 2- to 3-week follow-up. Macroscopic view of the resected RV-PA tissue revealed pannus formation particularly at the inflow (RV side) of the implanted LEAP Valve in these piglets (Fig. 7). Assessment of valve leaflet morphology and mobility by echo was limited due to acoustic shadowing from the stent. However, color Doppler imaging did not reveal regurgitation or paravalvular leak. Preliminary histological analysis revealed normal heart and lung tissues and no evidence of infection in the device, although the valve showed fibrin, immature fibrous tissue, and lymphocytes, plasma cells, and some multinucleated giant cells (fig. S7). The features of the cellular response were typical of a reaction to a foreign material. There was some leaflet thickening at 6 weeks. Using the Gorlin equation, we modeled the effect of reduction in valve effective orifice area due to pannus on the pressure gradient and found good agreement with our empirical measurements (table S5).

**Fig. 8.**
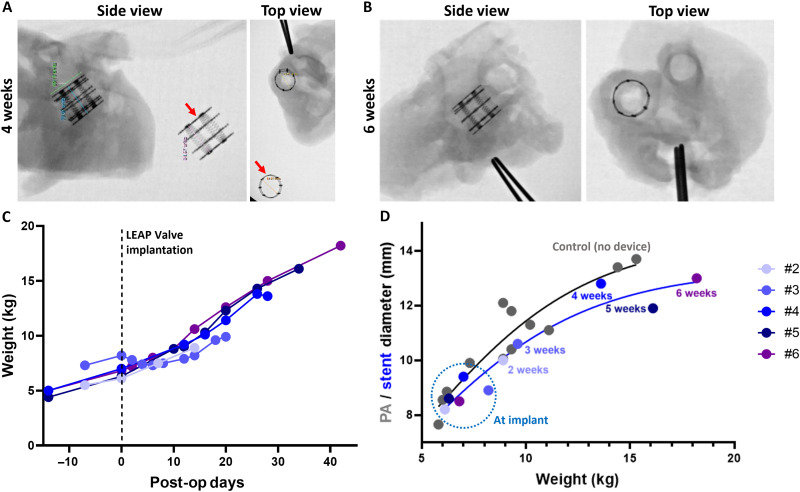
Noninvasive, spontaneous growth-adaptation of the stent. (**A**) X-ray images of the explanted heart with LEAP Valve at 4 weeks postimplantation. A fully expanded stent is shown in the x-rays for comparison (red arrows). (**B**) X-ray of LEAP Valve in the explanted heart after 6 weeks implantation. X-ray images show the uniform expansion of the stent. (**C**) Growth of piglets pre- and postimplantation. All piglets in the growth cohort gained weight. (**D**) Measurements of the LEAP Valve stent diameter at implantation (by echo) and termination (at explant: macroscopic photos for 2 and 3 weeks; x-ray images for 4, 5, and 6 weeks) for piglets #2 to #6 ranging 2- to 6-week follow-up. Piglet #1 is not shown because it was euthanized same day. PA diameter measurements by echo in piglets, which did not receive implants, are shown for comparison (gray), derived from a previous study (*36*).

Despite the formation of pannus, x-ray imaging of the explanted hearts showed that the function of the growth-adaptive stent was not restricted as the stents showed uniform expansion at 4 to 6 weeks (Fig. 8, A and B). Furthermore, we noted that, by echo at terminal surgery, the distal PA lumen measured 11.3 mm (5 weeks) and 13 mm (6 weeks), respectively, matching the stent outer diameters measured at explant (11.9 and 13 mm at 5 and 6 weeks, respectively). Overall, the stent diameter increased appropriately with growth over the course of 2 to 6 weeks that reasonably matched PA diameter growth in a separate cohort of nonimplanted animals (Fig. 8D). In addition, valve lumen diameter measurably increased in four of the five piglets, ranging ~9 to 17%, whereas the stent diameter increased 20% at 2-week follow-up (8.2 to 10 mm) and ~53% at 6 weeks (8.5 to 13 mm). Together, the piglet cohort demonstrated that the growth-adaptive mechanism of the stent functioned as designed by spontaneously increasing diameter with growth of the native PA.

## DISCUSSION

### Key achievements and potential impact

More than a quarter of babies born with CHDs have valve defects (*4*), which often require surgery for survival. Yet, there is no clinical standard for valve replacement in children under 5 years old and the options available to pediatric surgeons remain limited (*45*). Here, we describe a pediatric heart valve, the LEAP Valve, that spontaneously adapts to growth and is designed for young patients (neonates, infants, and toddlers) who are <20 kg in weight and who currently have no commercial prosthetic valve options <15 mm in diameter. The major accomplishments for this work and its implications are summarized below, followed by discussion of the remaining challenges and opportunities for this unique patient population.

#### Simple and tunable spring-based expansion mechanism of the stent

The key innovation of the LEAP Valve technology and focus of this work was the design of the growth-adaptive stent via a purely passive mechanical mechanism. Specifically, we exploited the superelastic properties of nitinol to create a simple low-force spring in the stent design that can spontaneously adapt diameter in the range of 7 to 14 mm (Figs. 1 to 3). This mechanism does not use the thermal-based shape memory property of nitinol commonly leveraged in other biomedical applications (*46*) and differs from stents that plastically deform at low strains (<0.5%), thus requiring diameter adjustment via a balloon catheter. The maximum target force requirement for our design in the fully compressed configuration of 7 mm was informed by our previous work characterizing the mechanical properties of piglet PA tissue (*36*) (fig. S1). Furthermore, we note the ability to adjust stent diameter range as well as target radial forces via spring thickness and/or number (*35*), making the concept readily tunable to other growth-adaptive needs for pediatric patients in the future.

#### Valve function at small diameters relevant to human neonates and infants

Using the rapidly growing Yucatan mini-swine as a model for the pediatric population of interest, we demonstrated that the LEAP Valve was initially constrained to the appropriate diameter (8.2 to 9.4 mm) and conformed to the lumen geometry of the main PA upon implantation. In addition, the device demonstrated good valve function (low gradient, no regurgitation) as well as spontaneous increase in valve diameter with growth for early follow-up at 2 to 3 weeks (Fig. 5). Piglet weights at implantation surgery and up to 2- to 3-week follow-up in the growth cohort ranged 6.1 to 9.6 kg, corresponding to human neonate through infant weight range (*19*) and valve diameter range (*13*, *19*). Overall, these findings support the feasibility of the LEAP Valve in meeting acute design and functional requirements for the youngest patients.

#### Spontaneous adaptation of stent diameter in a rapidly growing animal model

The growth cohort of five piglets terminated at 1-week intervals spanning 2 to 6 weeks postimplantation showed that the stent expanded proportionally along a growth curve, reaching 13 mm in diameter (50% increase) after 6 weeks (Fig. 8). All animals in the growth cohort gained weight postimplantation, and there were no device-related deaths or adverse events. Furthermore, no surgical or transcatheter interventions were used postimplantation of the LEAP Valve. The 6-week follow-up piglet reached a weight of 18.2 kg, which corresponds to a human child of about 5 years old (*19*). Now, neonates and infants with pulmonary stenosis or tetralogy of Fallot receive RV-PA conduits, which become increasingly dysfunctional and require surgical replacement after about 2 years (*47*, *48*). The benefits of eliminating even just one open-heart surgery include reduced risk of mortality in early life and improved outcomes (*49*). A 10-month-old infant implanted with a balloon-expandable valve will require a transcatheter procedure approximately every 10 months for the first few years of life (*15*). Although balloon expansion is less traumatic than surgery, it still has several limitations, including invasiveness of the procedure, uncertain timing of intervention relative to patient growth, and risk of damage to or infection of the valve with each intervention (*33*, *34*). Together, our initial results with the LEAP Valve stent represent an important first step toward a growth-adaptive device that can eliminate at least one surgical replacement of current commercial implants or one to three transcatheter procedures for balloon-expandable valves implanted in infants and toddlers (Fig. 1D).

### Remaining challenges

Developing a heart valve replacement for the youngest patients is a vision shared by many in the field. There have been numerous commendable and creative efforts to attain the holy grail of the “growing” valve through tissue engineering and expandable device designs (*22*, *27*, *29*–*31*, *50*). However, the unmet clinical need persists, at least in part, due to several challenges that have yet to be overcome. Accordingly, we acknowledge the lessons learned from our own work and that of others in the field. First, we note limitations of the animal model: Although sheep are recognized as the standard preclinical model for the evaluation of heart valve prosthetics, especially for longer-term studies (*51*), there are no established large animal models that truly represent the population of pediatric patients with CHD (*52*). We chose to use Yucatan mini-swine for our studies, due to their small size and weight range that matched the target pediatric population, rapid growth that allowed us to assess performance of the stent in a matter of weeks, and similar anatomy and tissue mechanical properties to pediatric patients (*36*, *53*). It will be important to address other biological questions related to LEAP Valve performance in vivo, potentially in multiple animal models, in future work.

We used the proven concept of the venous valve for the LEAP Valve to focus our efforts on the growth-adaptive stent. Although we achieved our primary goal of demonstrating that the stent appropriately and spontaneously adapted to growth in the piglets without intervention, as designed, we found that pannus formed at the valve inflow region at later time points (≥4 weeks), resulting in stenosis and increased transvalvular pressure gradients. On the basis of our preliminary histological findings, we concluded that the pannus formation was an inflammatory response to the human tissue graft that may have been exacerbated by the rapid growth and sensitive immune systems of the piglets (*51*). However, young children are also prone to pannus, which is a major cause of bioprosthetic valve failure in patients under 5 years old (*54*–*56*). This is an important challenge to address in future work—not only for the LEAP Valve but also for any heart valve design intended for infants and toddlers who grow rapidly. Clinical studies with valve graft materials, which have been used successfully in older children and adults, suggest that infants and young children have increased immunologic responses (*57*, *58*). Strategies to address inflammatory responses in future design iterations could include processing methods to reduce immunogenicity or improve endothelialization of the valve (*59*, *60*), the use of a different animal model such as the sheep or immunodeficient pigs (*61*), or even different valve materials—noting that our stent design itself is valve agnostic.

In addition to rapid growth, the small size and functional requirements for infant and toddler valve replacements present unique challenges. The thicknesses of engineered materials and tissues used in prosthetic valve designs cannot simply be scaled down for pediatric patients. The open orifice area of the valve decreases disproportionally faster than the diameter decrease, which can result in valves that have high outflow gradients at very small sizes. Our in vivo studies resulted in low gradients of <5 mmHg for functional valve diameters of ~8 to 9 mm upon initial implantation of the LEAP Valve. In contrast, our in vitro testing showed high gradients and smaller functional valve diameters that would not be acceptable in clinical use. We believe the discrepancies between our in vivo and in vitro results could be attributed to the stiffness of the device holders, which do not flex or allow expansion of the device with changing pressures as would happen in vivo, as well as variability in graft tissue thickness for the devices used in the animal cohort versus the in vitro tests. The ability to manufacture expandable valve materials that are consistently durable and thin to maximize effective orifice area over years of function will be key in the future. For example, there are several polymer-based PV replacements with low profiles at various stages of development (*27*, *31*, *62*). Again, recognizing that our stent design is valve agnostic, our work could synergize with other valve development efforts in the field.

### Limitations of the study and future opportunities

The main goal of this work was to demonstrate early preclinical feasibility of the spontaneous growth-adaptive mechanism of our stent design. As such, there are several limitations that should be addressed in the future. We limited our growth cohort to 6 weeks postimplantation to avoid the animals outgrowing the functional diameter of the device. However, durability and chronic host response will need to be evaluated for regulatory requirements, such as via accelerated wear testing and in good laboratory practice (GLP) studies. Chronic studies could present a hurdle for devices designed for young children that are intended for years of function in human patients but may be outgrown in preclinical animal models within weeks to months. In addition to identifying an appropriate animal model and time frame for growth-adaptive devices, tolerance for outgrowth and its effects on heart function will be an important question to address before trials in humans. Following further optimization of the valve material, detailed histological evaluation will be necessary to characterize host response to the implanted device. Although loss of effective orifice area due to pannus appeared to be a major contributing factor to the high pressure gradients observed, it will be important to consider other variables that can influence valve performance, such as calcification (*63*) and leaflet mobility (*64*) in future work. In addition, we did not investigate any potential RV or PA remodeling that may have occurred due to the forces exerted by the stent or high pressures that developed in some of the animals during the study. A better understanding of how the stent interacts with the surrounding tissue, and whether beneficial remodeling responses can be exploited while limiting adverse remodeling, could further improve the device design. We expect that these insights will derive from experimental and computational studies. The stent-tissue interaction model that we developed was simplified, and we further note that we have used the term “force” throughout this paper for its common use in the stent literature. We recognize that “stress” may be a more appropriate term to describe stent-tissue interactions. Modeling the localized stresses that result from stent-tissue interactions would be highly informative but is complex (*40*, *65*) and beyond the scope of the current work.

Despite the challenges and limitations noted above, there are a number of opportunities to pursue in the future that could have high impact for this young patient population. Our growth-adaptive stent design can be tailored for different diameter ranges, valve materials, and valve positions to provide solutions for a broader range of pediatric patients who have valve defects. Although our initial focus is PV replacement as it is lower risk compared to left heart applications, there remains a great need for pediatric aortic and mitral valve replacements. We also postulate that the stent could be used as a standalone technology for other pediatric indications in which a growing lumen is desired, such as in vascular, intestinal, or urinary tract congenital defects. Overall, the spontaneous growth-adaptive stent concept could provide advantages over balloon-expandable devices by avoiding multiple invasive procedures and associated complications.

In summary, we have presented a feasibility study describing a rationally designed stent that spontaneously adapts to growth through a springlike mechanism without the need for intervention after implantation. Our stent design has the potential to change the standard of care for pediatric patients who need technologies that can accommodate their rapid growth. We envision several impactful benefits of this technology: providing a viable valve prosthetic option for neonates, infants, and toddlers; eliminating surgeries and other invasive procedures in the early years of life; and providing a ready-to-use, accessible technology that does not require modifications for implantation or regular hospitalizations for specialized expansion procedures as children grow.

## MATERIALS AND METHODS

### Growth-adaptive stent design and FEA

To achieve the desired twofold diameter expansion range, the LEAP Valve stent was designed in nitinol, a superelastic alloy that allows for up to 8% strain without plastically deforming (*66*) (Fig. 2A). Nitinol was also chosen for its biocompatibility, ease of manufacturing, and prior history in other stent designs (*66*, *67*). ANSYS 2021 (Canonsburg, PA) FEA software was used to simulate different stent designs. Several features were implemented in the final stent design: (i) a constant stent length of 15 mm with diameter change through eight equally spaced longitudinal struts, (ii) clocking features on the struts to track stent segments for dimensional characterization (*39*), (iii) suturing features along the struts for valve attachment, and (iv) the spring elements that provide the expansion mechanism for the stent (Fig. 2B). The FEA leveraged stent rotational symmetry by simulating the geometry of a single spring as shown in Fig. 2C. The curved spring geometry was imported into a static structural analysis that incorporated both geometric and material nonlinearities. The outer diametrical surface of the curved spring was radially displaced from the nominal 14-mm diameter to a 7-mm diameter and then unloaded back to a 14-mm diameter. The maximum radial reaction force resulting from the displacement was computed. The predicted total stent radial force was calculated by multiplying the simulated radial reaction force per spring by the number of desired springs per stent. Simulated designs that were at or below the maximum radial force target and could achieve the twofold diameter change with less than 8% strain were then manufactured. On the basis of our previous work characterizing the properties of piglet PA and valve annulus tissue (*36*), as well as previous studies of maximum outward radial force thresholds for self-expanding nitinol stents (*40*, *41*), the final stent design used in the animal studies exerted a maximum radial force of ~7 N at its smallest diameter of 7 mm.

### Growth-adaptive stent manufacturing

The LEAP Valve stent was manufactured at Resonetics (San Diego, CA) using femtosecond laser cutting capabilities. The manufacturing process started with a 7-mm-diameter, 0.33-mm-thick stock tubing of nitinol (ASTM F2516, G.RAU Innovative Metalle, Germany). The stent design in the compressed configuration was ablated into the stock tube. Next, the stents underwent a series of heat treatment steps to gradually increase the outer diameter from 7 to 14 mm. After the final heat treatment, the zero-stress state of the stent was set at its fully expanded diameter of 14 mm. Last, the stents underwent an electropolishing step to remove any burrs produced by laser cutting and to improve biocompatibility for animal studies.

### Radial force testing

Once manufactured, all stents received a tracking number and were imaged using a Keyence VHX5000 (Osaka, Japan) digital microscope. We then used our custom image processing software to measure important design features of the stent (*39*) and to capture any manufacturing anomalies. Next, each stent was tested to characterize radial force behavior (Machine Solutions Inc., Flagstaff, AZ) using a RX550/650, 12-point contact radial compressor through six cycles at a compression/expansion rate of 0.05 mm/s at 37°C. Afterward, the stents underwent a second round of imaging on the Keyence system to determine whether plastic deformation occurred during radial force testing. This was determined by measuring the fully expanded diameter before and after radial force testing to check for any significant changes. The stents that produced the most consistent radial force curves up to the desired maximum force of 7 N were selected for animal studies (Fig. 3D and table S1).

### Implantation model: Stent-tissue interaction

Given that the stent imparts the greatest force at its smallest compressed diameter of 7 mm, we sought to model tissue deformation at the implantation site in the main PA as well as the anticipated stent force at the equilibrium state, once the outward radial force of the stent balanced with the restraining force of the surrounding native tissue. To this end, we developed an analytical implantation model based on the mechanical properties of piglet PA tissue characterized in our previous work (*36*) and the experimental radial force data from our manufactured stents described above. The original, raw PA tissue dataset was a pressure versus diameter curve, where the applied pressure was converted to tissue stress via the thin-walled hoop stress equation and the diameter was converted to strain. A stress-strain curve was generated from which the Young’s modulus of the PA tissue was calculated at different levels of stress ranging from physiological to supraphysiological loading conditions. In the analytical model, we determined the restraining force from the tissue as follows. Strain was applied from an initial diameter of 7 mm, and the amount of stress to yield the corresponding Young’s modulus from the empirical data was computed. The resulting stress was then decomposed into the hoop stress imparted by a representative blood pressure of 15 mmHg (approximate mean pressure in the PA) and the stress caused by an applied stent force (computed by dividing the stent force by the surface area in contact with the PA). The stent force was then plotted against different diameters, representing for a given diameter, how much applied force would be needed to achieve it. The average outward radial stent force data, unloading curve only (i.e., expanding stent condition), and calculated tissue restraining force of the PA were plotted against each other, assuming an initial diameter of 7 mm. To capture anticipated biological variability, a range of restraining forces from the PA tissue was plotted on the basis of empirical tissue stiffness values 1 SD above and below the mean (fig. S1). The intersection of the stent force curve with the tissue restraining force curve indicated the anticipated equilibrium for stent force and PA/stent diameter.

### Radial compressor tool

A custom handheld radial compressor tool was designed to compress the growth-adaptive stent for simple bench tests of LEAP Valve competency and to aid animal implantation studies (figs. S2 and S3). The radial compressor tool consisted of two 316 stainless steel waterjet cut guides with 3D printed titanium tangs that formed an iris mechanism to symmetrically compress the stent for surgical preparation. The radial compressor tool was designed with a handle hold that allowed the user to compress or expand the iris mechanism across a functional range of 5 to 20 mm in diameter. The whole assembly was made from autoclavable material for sterilization.

### LEAP Valve device assembly

Commercially available cryopreserved HFV grafts (Artivion, formerly CryoLife) were used as the adaptable venous valve source for the device. Valves were selected from the grafts based on the maximum outer diameter of the valve when pressurized (15 to 17 mm), bileaflet geometry, and a commissure height of ≤15 mm (table S2). Before valve attachment, the growth-adaptive stents were sterilized by 24-hour exposure to ethylene oxide gas at low temperatures in an Andersen Anprolene AN74j sterilizer. HFV grafts were provided by the manufacturer in three-layer packaging with a proprietary solution that contains antibiotics. We used a clean space with negative air vacuum during device integration and suturing. The working area was covered with sterile drapes and sterile instruments, and standard sterile surgical gowns, gloves, and masks were used for all steps to maintain sterility of the device during assembly.

First, valve size, alignment, and competency were checked by pressurizing the HFV using a syringe and the manufacturer’s tissue preservation solution. Then, the selected valve area was placed inside the stent, and the graft was reinflated with backpressure to expand the valve. At full expansion of the valve, we ensured the following: (i) The valve was able to expand flush against the entire circumference of the stent, (ii) the valve height from the sinus to the commissures fit within the stent length of 15 mm, and (iii) no leak was observed. Once these criteria were confirmed, the excess graft tissue was trimmed and removed. The remaining vein segment with the selected valve attached to the syringe was then expanded with the solution to maintain the valve geometry while anchoring to the stent.

To anchor the valve to the growth-adaptive stent, we used 6-0 prolene suture (Ethicon EP8709H, Somerville, NJ, USA). A simple interrupted suture was placed at select anchoring points at the proximal end, distal end, and in the middle of each of the eight stent struts, for a total of 24 sutures to attach the valve. The sutures at the proximal and distal end of each strut were full-thickness bites of the vein to provide positional anchoring, whereas the sutures at the middle of the strut were more superficial and aimed to only attach the adventitial surface to the stent. The distal and proximal positions of the same strut were always sutured sequentially to prevent twisting. The second strut sutured was directly opposite the first strut, and subsequent struts were sutured in an alternating pattern. Extreme care was taken to ensure that no suture pierced the valve commissures. After all three positions of all eight struts were sutured, the excess vein tissue was trimmed to the size of the stent.

To confirm the function of the assembled LEAP Valve, we repeated the competency test with saline and inspected for any leaks. In addition, we inspected the stent to ensure that there was no distortion in its shape or valve damage. Once the device check was completed, we stored the LEAP Valve in a sterile tube with sterile phosphate-buffered saline (PBS) solution. The device was then shipped overnight on ice to the Seattle Children’s Research Institute for the animal studies. Upon arrival, the sterile PBS solution was exchanged, and the LEAP Valve was kept in a storage refrigerator at 4°C. Typically, we completed the assembly and shipping 48 hours before the surgery to ensure that the device arrived on time and to minimize the risk of infection or valve deterioration from assembly to surgical implantation.

### In vitro performance

Two additional LEAP Valve devices were assembled as described above and then subjected to hydrodynamic performance testing. The valves were tested across four flow conditions, corresponding to normal and high cardiac outputs for neonate (8.5-mm stent diameter) and 2-year-old toddler (14-mm stent diameter) pediatric subpopulations. For both patient groups, the valves were deployed at low-normal diameters of 8.5 and 14 mm, respectively, to represent the slower growth that the population of patients with CHD of interest may follow. The diameter of the valve lumen after deployment but before pressurization was measured using ImageJ. Normal cardiac output for each group was defined as 3 liters/min per square meter, normalized to the body surface area (BSA) for each patient size. Normal heart rate for each BSA was taken from the Boston Children’s Hospital *z*-score database (*68*). High cardiac output was 4 liters/min per square meter, with stroke volume held constant from the normal cases, consistent with the literature for pediatric exercise response (*69*). Detailed test conditions are provided in table S3. Hydrodynamic testing was carried out in a pulse duplicator (ViVitro Labs, Victoria, BC, Canada) to determine transvalvular pressure gradient, regurgitation, and leaflet dynamics for each of the conditions. Tests were conducted with 0.9% sodium chloride solution. Valves were deployed within custom silicone holders and secured with two (8.5-mm diameter) or four (14-mm diameter) sutures on the ventricle side to prevent device migration (fig. S4). In the 14-mm holders, a thin layer of silicone adhesive was applied to the proximal end of the stent to mimic tissue ingrowth and prevent perivalvular flow. Mounted valves were placed in the outflow position within the pulse duplicator system. Average measurements over 10 cycles are reported.

### Overview of animal studies

A total of 10 piglets (Yucatan mini-swine, Premier BioSource, CA) were used for acute implantation studies (*N* = 4) and the growth cohort (*N* = 6) as described in further detail below. All animal procedures were performed at Seattle Children’s Research Institute. All procedures for these animal experiments were conducted according to the National Institute of Health’s Guide for the Care and Use of Laboratory Animals and were reviewed and approved by Seattle Children’s Institutional Animal Care and Use Committee (IACUC, protocol no. ACUC00633) and the US Army Medical Research and Development Command’s Animal Care and Use Review Office (ACURO).

### Development of the device implantation method

We developed the LEAP Valve implantation method by using ex vivo pig hearts and in vivo nonsurvival acute surgeries (*N* = 4 piglets; 36 to 69 days old, 5.0 to 10.6 kg). Anesthesia, mechanical ventilated support, and median sternotomy were performed as described previously (*70*). Several positions between the native PV annulus and the main PA were considered for device implantation, with final selection in the proximal main PA. We avoided the RV muscles due to different tissue characteristics and contractile movement that could affect LEAP Valve function. We started with placing two to four anchoring sutures on the proximal end of the stent. Then, we compressed the LEAP Valve device, using the radial compressor tool described above, to its minimum diameter of 7 mm and secured it with a restraining suture loop. Although the piglet was anesthetized and on CPB, we opened the PA and resected the native PV leaflets. We then passed the anchoring sutures on the LEAP Valve from inside to outside the vessel and kept them loose. Following that, we placed the compressed LEAP Valve within the PA lumen and began closure. Before tightening the last few sutures to fully close the PA, we removed the restraining suture loop around the LEAP Valve to allow the stent to conform to the vessel geometry. After completing the PA closure, the anchoring sutures were tightened. This workflow sped up our implantation process and reduced the implantation time to less than 30 min.

### Survival studies in growing piglets

For survival studies, we performed implantation of the LEAP Valve into the main PA of *N* = 6 live Yucatan mini piglets (46 to 56 days old, 6.1 to 8.2 kg). In vivo LEAP Valve implantation was performed under beating heart conditions with CPB support, as described in detail below. One piglet was euthanized on the day of surgery at 2.5 hours postimplantation, whereas two piglets were observed for 2 and 3 weeks after valve implantation. Next, we investigated the growth-adaptation mechanism of the LEAP Valve stent at predetermined time points of 4, 5, and 6 weeks (*N* = 1 at each time point).

Piglets were initially sedated with an intramuscular injection of ketamine (33 mg/kg), xylazine (2 mg/kg), and atropine (0.02 mg/kg). The animals were intubated with a cuffed endotracheal tube, facilitating mechanical ventilation and general anesthesia with inhaled isoflurane (1 to 3%). Electrocardiogram, oxygen saturation (SpO_2_), end tidal CO_2_, and rectal temperature were recorded. Antibiotic (Excede, 0.05 ml/kg) and analgesia (ketoprofen and buprenorphine) were intramuscularly administered. Angiocatheters were inserted into the femoral artery and the external jugular vein for continuous recording of the pressure, fluid infusion, and blood gas measurement. Access to the heart and PV was achieved by performing a left anterolateral thoracotomy in the fourth intercostal space. CPB was established with 3.5-inch mast mounted roller pump (MRP 85; Liva Nova, Arvada, CO) and oxygenator (KIDS D100; Liva Nova, Arvada, CO) including a 3/16-ID pump boot and 3/16-ID x 1/4-ID arterial/venous lines (Terumo Cardiovascular, Ann Arbor, MI). The venous reservoir was positioned level with animal height using vacuum-assisted venous return. The total circuit prime volume was 85 ml. After systemic heparinization (350 IU/kg) and cannulation via the ascending aorta (8Fr DLP; Medtronic, Minneapolis, MN) and right atrium (18Fr DLP; Medtronic), full flow bypass was established at a 2.2 Cardiac Index and piglets were cooled to 32° to 34°C. Under beating heart conditions, a longitudinal incision of the main PA was made, and native PV leaflets were removed. The LEAP Valve was compressed to 7 mm in diameter for implantation as described above. LEAP Valves were inserted and sutured in place with two anchor stitches, which was determined from the acute implantation studies as the minimum number required to keep the LEAP Valve secured without migration or paravalvular leak. After device implantation, the PA was closed. Piglets were weaned off CPB, heparin was neutralized by protamine, and the cannulas were then removed. The endotracheal tube was removed once the animal had awakened. Once deemed stable, the animal was moved to the postoperative care area for further recovery. We gave low-molecular-weight heparin (1 mg/kg, twice per day) via subcutaneous injection as an anticoagulation therapy and suitable analgesia for 3 days after surgery. We did not use additional anticoagulation or immunosuppressive therapies for these studies.

### Assessment of acute function and growth-adaptation of the LEAP Valve in piglets

A direct echocardiogram on the epicardium was performed using a GE Vivid I ultrasound machine (GE HealthCare Technologies Inc., Chicago, IL) to measure the PA diameter and valve function at the following time points: (i) before LEAP Valve implantation as baseline data, (ii) after the weaning off of CPB to measure acute function and diameter of the LEAP Valve immediately after implantation, and (iii) at the designated follow-up assessments at terminal surgery. We collected pressure measurements in the PA and RV by pressure catheter insertion in addition to continuous recording of arterial pressure and electrocardiogram. PAP and RVP measurements were made individually, not simultaneously, and then averaged over 10 cardiac cycles. Terminal surgery at the scheduled follow-up was performed under full anesthesia, and the implanted LEAP Valve was evaluated with epicardial echocardiography and pressure measurement after reaching the heart and large vessels through a median sternotomy. Following terminal surgery measurements, the animals were euthanized and the heart with PA was explanted. The device was evaluated macroscopically after the resection of the RV outflow tract and PA en bloc. For the 2-week time point, the LEAP Valve was extracted and examined in detail. For the 4-, 5-, and 6-week time points, the explanted heart was first imaged by x-ray and then fixed in formalin for histology. Stent outer diameter was measured during macroscopic evaluation for the 2- and 3-week follow-up and by x-ray imaging of explanted hearts for piglets with 4-, 5-, and 6-week follow-up after LEAP Valve implantation, as presented in Fig. 8D and Table 2.
