# Supplementary material for: Design and preclinical feasibility of a pediatric heart valve stent that spontaneously adapts to growth via a spring mechanism
Source: Sci Adv. 2026 Feb 18;12(8):eadw4669. doi: 10.1126/sciadv.adw4669 (PMC12915617; doi:10.1126/sciadv.adw4669)
Supplement: Supplementary file 1 — Supplementary Text Figs. S1 to S7 Tables S1 to S5 [file sciadv.adw4669_sm.pdf]

Science Advances
AAAS

Supplementary Materials for

Design and preclinical feasibility of a pediatric heart valve stent that spontaneously adapts to growth via a spring mechanism

Giselle Ventura *et al.*

Corresponding author: Corin Williams, cwilliams@draper.com;
Michael A. Portman, michael.portman@seattlechildrens.org; Sitaram M. Emani, sitaram.emani@cardio.chboston.org

Sci. Adv. **12**, eadw4669 (2026)
DOI: 10.1126/sciadv.adw4669

This PDF file includes:

Supplementary Text
Figs. S1 to S7
Tables S1 to S5

Supplementary Text

MATERIALS AND METHODS

Anatomical measurements of pulmonary valve and artery in healthy Yucatan mini pigs

We performed an initial set of experiments to measure the PA size in healthy piglets. Measurements of normal PA dimensional ranges were fundamental to the identification of the appropriate piglet size and growth rate. Twelve healthy Yucatan mini pigs (40-56 days old, 5.8-8.2 kg; Premier BioSource, CA) were used for *in vivo* anatomical measurements of the PA to estimate growth projections. These data included measurements that we had previously made, reported in Sutherland & McEleney (2022) (36), and the growth cohort in this study, in which measurements of the PA diameter were made immediately prior to implantation of the LEAP Valve. After appropriate anesthesia, mechanical ventilated support and median sternotomy was performed as described previously (70) and in the main manuscript. Direct echocardiogram on the epicardium was performed to measure the diameter of the PA lumen by using a GE Vivid I ultrasound machine (GE HealthCare Technologies Inc., Chicago, IL). PA diameter measurements were then plotted as a function of piglet age or body weight.

Characterization of Nitinol pre- and post-heat treatment

The growth-adaptive stents were cut in Nitinol tubing at 7 mm diameter, expanded, and then heat treated to set the zero-force, fully expanded outer diameter of 14 mm. To quantify the effect of heat treatment on stent performance, Nitinol tubing material characterization was performed. Six tensile-test coupons were laser-cut (Resonetics, San Diego, CA) out of 7 mm diameter Nitinol tubing (ASTM F2516, G.RAU Innovative Metalle, Germany). Three of the six coupons were heat treated. Tensile testing (Metallurgical Engineering Services Inc., Richardson, TX) was performed on all six test specimens per ASTM E8-21 using a Satec Systems Model Apex 22E, and stress-strain curves were generated (***Figure S6***).

Histology

For the piglets that had high pressure gradients and pannus formation at 4, 5, and 6 weeks post-implantation of the LEAP Valve, we performed preliminary histological analysis (University of Minnesota, Experimental Surgical Services; StageBio). The RVOT and PA containing the implanted LEAP Valve was extracted en bloc and fixed in formalin. Heart and lung samples were also collected, embedded in paraffin, and stained with hematoxylin and eosin. The LEAP Valve samples were dehydrated in a graded series of ethanol, then infiltrated with and embedded in SPURR plastic resin. After polymerization, the samples were sectioned and stained with hematoxylin and eosin.

Modeling of the pressure gradient due to pannus formation

For the piglets that had high pressure gradients and pannus formation at 4, 5, and 6 weeks post-implantation of the LEAP Valve, we modeled the anticipated contribution of pannus (in terms of reduced valve orifice area) on the pressure gradient. First, we used the X-ray images to measure the valve inner diameter and calculated the percent reduction in effective orifice area. We then used the Gorlin equation to calculate the pressure gradient as follows:

$$PG = \left[\frac{\mathrm{CO} * 1000\frac{\mathrm{mL}}{\mathrm{L}}}{44.3 * \mathrm{T_{SF}} * \frac{60\mathrm{s}}{\mathrm{min}} * \mathrm{A}}\right]^2$$

where:

PG = pressure gradient in mmHg

CO = cardiac output in liters per minute (see calculations below)

T_{SF} = Systolic fraction time (set to 0.34, close to physiological value)

A = area of the valve in cm^2 (measured from the X-ray images, see Table S5)

To determine cardiac output, we assumed that pigs and humans of same weight have similar cardiac output. We used CDC growth curves for boys to find age for a given weight. We then used BCH's database to find body surface area for a given age. BSA and cardiac index of 3 LPM/m^2 was used to calculate CO in LPM:

13.6 kg = 29 mo = 0.55 m^2 x (3 LPM/m^2) = 1.65 LPM

16.1 kg = 4 yrs = 0.7 m^2 x (3 LPM/m^2) = 2.1 LPM

18.2 kg = 5 yrs = 0.79 m^2 x (3 LPM/m^2) = 2.4 LPM

Using CO values, T_{SF}, and valve area measurements for the 4, 5, and 6 week piglets in the equation above resulted in the PG values presented in ***Table S5*** for comparison against our empirical measurements by Doppler echo.

RESULTS

Development of the implantation strategy for the LEAP Valve

Implantation strategy for the LEAP Valve was developed in 4 piglets. For the first piglet (5.5 kg), the middle of the LEAP Valve was secured at the pulmonary valve (PV) annulus with 4 anchoring sutures, such that the proximal end of the device was in the right ventricle (RV), while the distal end was within the PA. We found that the diameter at the distal end of the stent was larger than at its proximal end, indicating coning behavior of the stent in this position. The longitudinal struts of the growth-adaptive stent are designed to provide stability and support symmetrical expansion of the valve for optimal function. We hypothesized that non-uniform expansion of the stent in the PV annulus position could be due to differences in native tissue mechanical properties at different anatomical locations (e.g., RV, PV annulus, PA). Therefore, an implantation site with relatively uniform mechanical properties and structure, such as the main PA, was deemed best for the LEAP Valve design.

For the second piglet (5 kg), the LEAP Valve was implanted in the main PA with 4 anchoring sutures. However, we found that this number of anchoring sutures was complicated due to limited visibility within the main PA compared to the PV annulus. In addition, the main PA, which naturally curves, was only ~15 mm in length and caused the device to buckle. From here, we decided to use fewer anchoring sutures to reduce complexity of the procedure and larger piglets

with main PA length ≥18 mm to ensure sufficient straight anatomy section to accommodate the LEAP Valve.

For the third and fourth piglets (7.3 kg and 10.6 kg, respectively), the LEAP Valve was implanted into the main PA of length >18 mm with only 2 anchoring sutures. We note that pigs 1, 3, and 4 were weaned off of CPB smoothly; the second pig did not recover due to device buckling as described above. The fourth piglet was recovered from surgery for approximately 2.5 hr prior to termination on the same day. Successful implantation with the third and fourth pigs led us to adopt the strategy of 2 anchoring sutures and positioning of the LEAP Valve in the proximal main PA for subsequent survival studies.

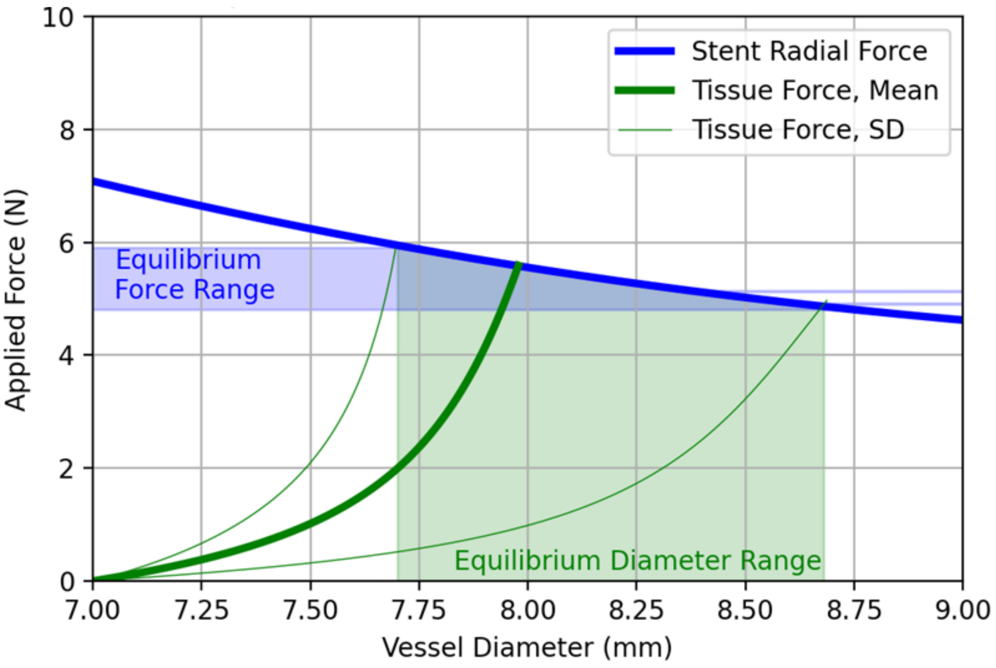

Figure S1: Stent-tissue interaction model. Average stent radial force as a function of diameter is plotted against empirical data for piglet PA tissue deformation, derived from Sutherland & McEleney et al (2022) (36), to estimate the diameter change with force equilibration upon implantation of the compressed LEAP Valve. The worst-case scenario of 7 mm diameter is shown, at which the stent exerts its maximum force. The intersection between the stent force and counteracting tissue force indicates an average diameter at equilibrium of 7.9 mm (range: 7.7 – 8.6 mm), or an average increase of 13% (range: 10-23%) from the initial diameter.

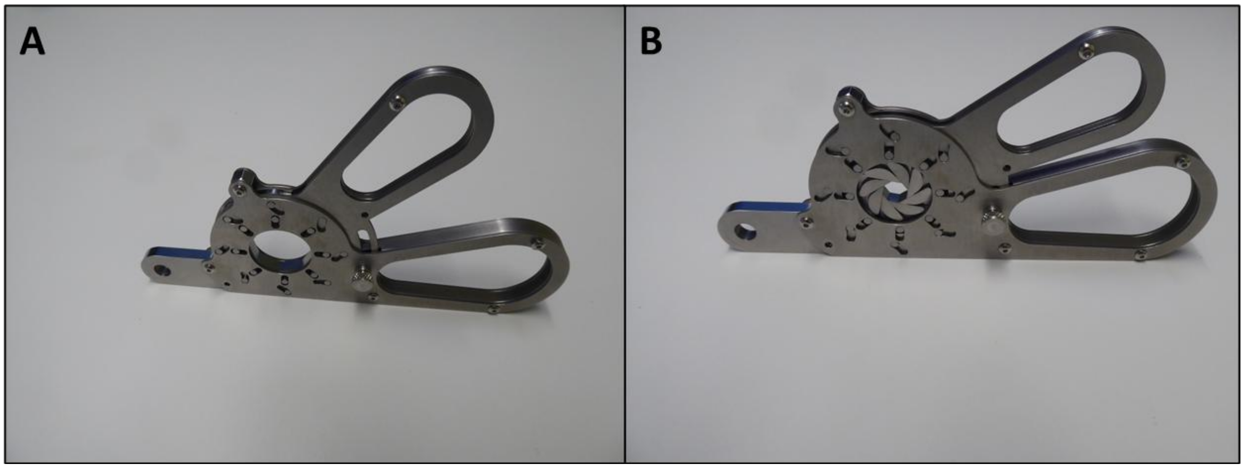

Figure S2: Custom Radial Compressor Tool. (**A**) Demonstrates the radial compressor in the fully open state. (**B**) Demonstrates the radial compressor in the closed state that would symmetrically compress the growth-adaptive stent to 7 mm diameter. The thin profile of the radial compressor tool provides access to both ends of the stent to apply restraining sutures for implantation of the compressed LEAP Valve.

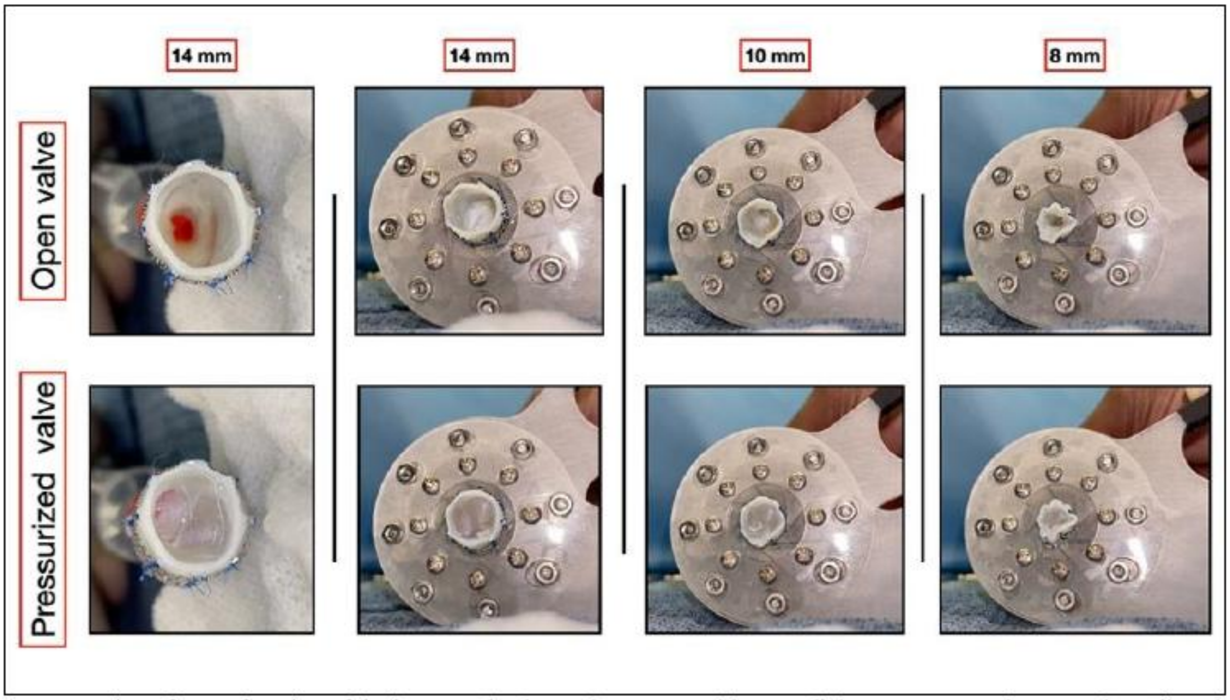

Figure S3: Acute function/ bench testing of the LEAP Valve. An assembled LEAP Valve is shown in the open and closed states at different diameters.

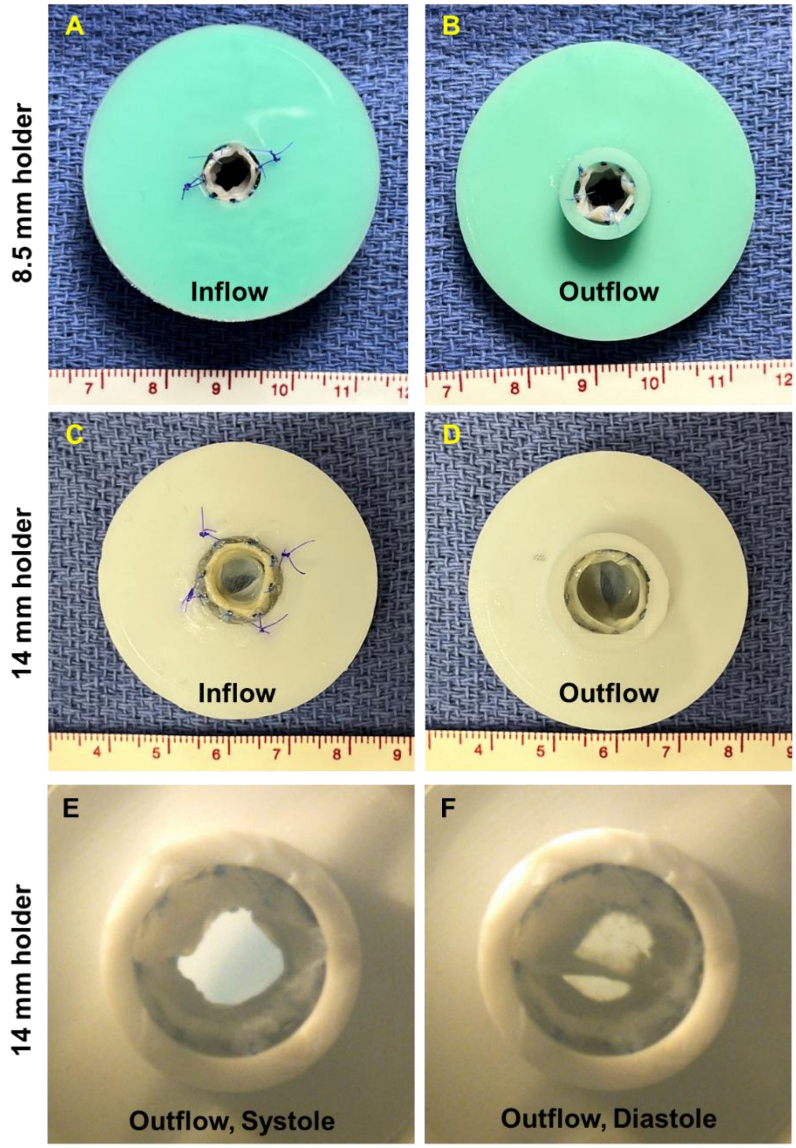

Figure S4: LEAP Valves for *in vitro* hydrodynamic testing. (**A,B**) Top row shows the device deployed into the neonatal size 8.5 mm diameter holder. (**C, D**) Bottom row shows the device in the infant/toddler size 14 mm diameter holder. Left column shows the ventricle (inflow) side of the holder with anchor sutures visible. Right column shows the PA (outflow) side, with closed leaflets visible in (D). Representative images of the LEAP Valve under hydrodynamic testing in the pulse duplicator system, outflow view in (**E**) systole and (**F**) diastole.

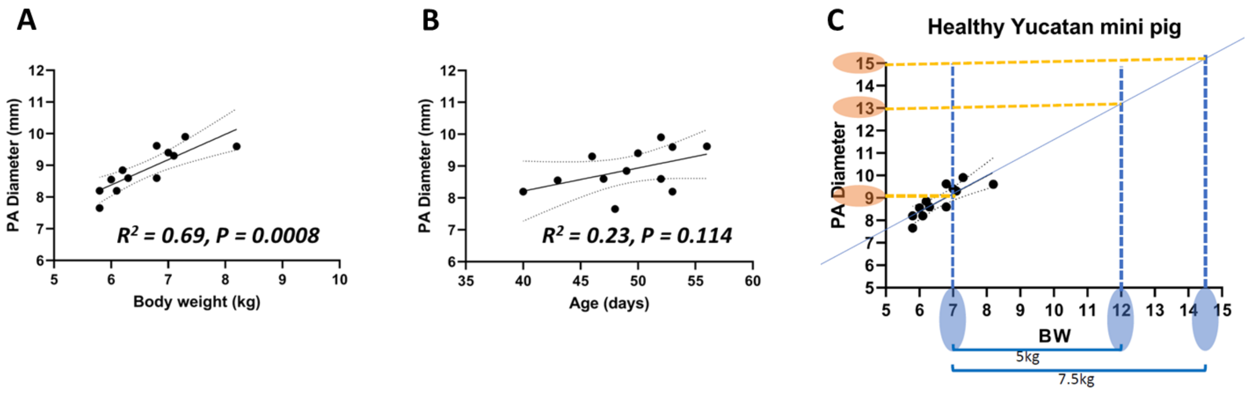

Figure S5: Piglet growth projections. (**A**) PA diameter as a function of piglet weight. (**B**) PA diameter as a function of piglet age. (**C**) Projected growth of piglets to target weight. Approximate tripling in weight corresponds to approximate doubling in valve diameter. Estimated ideal follow-up post-implantation was determined to be 4-6 weeks based on weekly weight gain.

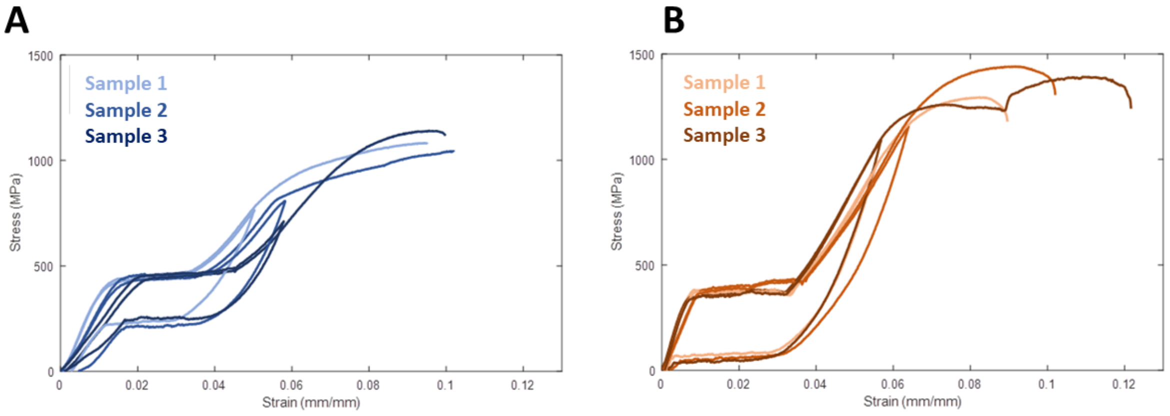

Figure S6: Stent material characterization. Stress-strain curves for Nitinol stent tubing material (A) as cut, prior to heat treatment and (B) after heat treatment.

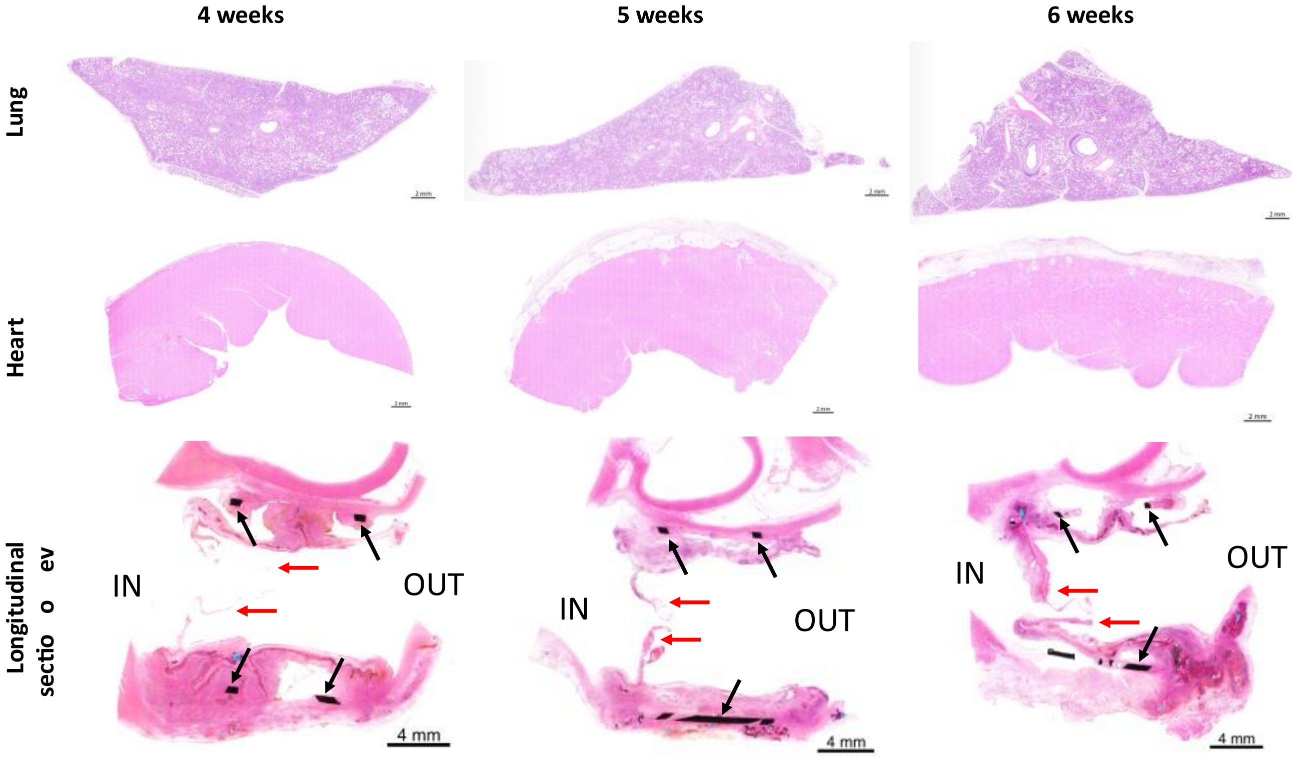

Figure S7: Histology. Explanted lung and heart tissues and LEAP Valve devices within the main PA at 4, 5, and 6 weeks post-implantation were sectioned and stained with H&E. Black arrows indicate portions of the stent. Red arrows indicate leaflets. “IN” and “OUT” indicate the inflow and outflow regions, respectively. Note that these histology sections correspond to the same devices shown in Figure 7.

Table S1. Stents used for animal studies

Stent No.	*Max Radial Force [N] at 7 mm Diameter*	*Study Used*
12	7.1	Acute Study, Piglet 1
16	7.4	Acute Study, Piglet 2
20	6.9	Acute Study, Piglet 3
17	7.6	Acute Study, Piglet 4
5	7.4	Growth Cohort, Piglet 1
13	7.3	Growth Cohort 1, Piglet 2
23	7.6	Growth Cohort 1, Piglet 3
18	6.9	Growth Cohort 1, Piglet 4
14	6.9	Growth Cohort 1, Piglet 5
8	7.1	Growth Cohort 1, Piglet 6

Table S2: Assessment of commercial venous valve sources

	Human femoral vein	***Bovine jugular vein***
Size range ID	8 to 14 mm	12 to 22 mm
Size range OD	9 ± 0.5 to 16 ± 0.5 mm	13 ±1 to 23 ±1 mm
Commissural height	15 to 20 mm	Minimum 20 mm
Number of leaflets	Two	Three
Advantages	- Low profile<br>- Has shorter commissure height<br>- Both leaflets move with downsizing<br>- Good source for small valve diameters<br>- Short valves available<br>- Less variability in valve geometry and function at baseline	- Good source for larger valve diameters
Disadvantages	- Size/diameter limit	- Thick wall<br>- Higher commissures<br>- Leaflet movement restriction with downsizing<br>- Valve function and geometry variability at baseline
Example images	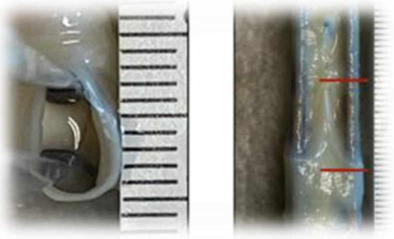	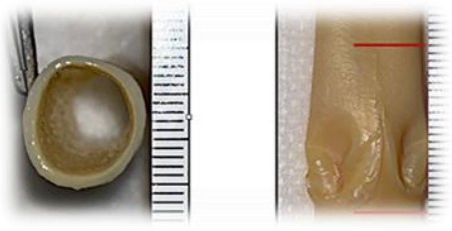

Table S3: Hydrodynamic flow parameters for in-vitro valve testing

	**Neonate**	**Toddler (2 years)**
Body surface area (m^2)	0.243	0.563
Normative pulm annulus diameter (mm)	9.8	15
Deployed LEAP Valve outer diameter (mm)	8.5	14
**Normal cardiac output test condition**		
Cardiac index (L/min/m^2)	3	3
Heart rate (cycles/min)	128	110
Systolic duration (% of cycle)	50	45
Stroke volume (mL)	5.7	15.4
Mean arterial pressure target (mmHg)	20	20
**High cardiac output test condition**		
Cardiac index (L/min/m^2)	4	4
Heart rate (cycles/min)	171	147
Systolic duration (% of cycle)	50	50
Stroke volume (mL)	5.7	15.4
Mean arterial pressure target (mmHg)	20	20

Table S4: Results from *in vitro* hydrodynamic valve testing.

	Neonate		Toddler (2 years)	
	**Device 1**	**Device 2**	**Device 1**	**Device 2**
**Normal cardiac output test condition**				
Pressure gradient (mmHg)	67 ± 1	52 ± 1	26 ± 1	20 ± 1
Regurgitation (%)	14.6 ± 1.0	6.0 ± 0.4	7.0 ± 0.7	6.5 ± 0.8
**High cardiac output test condition**				
Pressure gradient (mmHg)	100 ± 1	77 ± 1	39 ± 1	28 ± 1
Regurgitation (%)	6.4 ± 0.7	5.0 ± 0.5	6.2 ± 0.4	4.3 ± 0.5

Reported values are mean ± standard deviation over 10 cycles.

Table S5: Modeling of the Pressure Gradient

Pig #	Weight (kg)	Stent OD (mm)	Valve ID (mm)	Reduction in Orifice Area (%)	PG by Gorlin Eqn. (mmHg)	PG by Echo (mmHg)
2	8.9	10	--	--	--	6
3	9.6	10.6	--	--	--	5
4	13.6	12.8	6.4	75	32	28-30
5	16.1	11.9	7.1	64	34	23-25
6	18.2	13	8.8	53	19	13-15

Stent outer diameter (OD) and valve inner diameter (ID) were measured from X-ray images of the explanted piglet hearts at 4, 5, and 6 weeks post-implantation. PG = pressure gradient.
